# Pyrene Coating Transition Metal Disulfides as Protection from Photooxidation and Environmental Aging

**DOI:** 10.3390/nano10020363

**Published:** 2020-02-19

**Authors:** Ruben Canton-Vitoria, Yuman Sayed-Ahmad-Baraza, Bernard Humbert, Raul Arenal, Christopher P. Ewels, Nikos Tagmatarchis

**Affiliations:** 1 Theoretical and Physical Chemistry Institute, National Hellenic Research Foundation, 48 Vassileos Constantinou Avenue, 11635 Athens, Greece; canton@eie.gr; 2 Institut des Materiaux Jean Rouxel (IMN), UMR6502 CNRS, Universite de Nantes, 2 Rue de la Houssiniere, BP32229, 44322 Nantes, France; yusabar@gmail.com (Y.S.-A.-B.); bernard.humbert@cnrs-imn.fr (B.H.); 3 Laboratorio de Microscopias Avanzadas, Instituto de Nanociencia de Aragon, Universidad de Zaragoza, 50018 Zaragoza, Spain; 4 ARAID Foundation, 50018 Zaragoza, Spain; 5 Instituto de Ciencias de Materiales de Aragon, CSIC-U. Zaragoza, 50009 Zaragoza, Spain

**Keywords:** MoS_2_, WS_2_, 2D materials, exfoliation, functionalization, pyrene, oxidation, aging, protection, DFT calculations

## Abstract

Environmental degradation of transition metal disulfides (TMDs) is a key stumbling block in a range of applications. We show that a simple one-pot non-covalent pyrene coating process protects TMDs from both photoinduced oxidation and environmental aging. Pyrene is immobilized non-covalently on the basal plane of exfoliated MoS_2_ and WS_2_. The optical properties of TMD/pyrene are assessed via electronic absorption and fluorescence emission spectroscopy. High-resolution scanning transmission electron microscopy coupled with electron energy loss spectroscopy confirms extensive pyrene surface coverage, with density functional theory calculations suggesting a strongly bound stable parallel-stacked pyrene coverage of ~2–3 layers on the TMD surfaces. Raman spectroscopy of exfoliated TMDs while irradiating at 0.9 mW/4 μm^2^ under ambient conditions shows new and strong Raman bands due to oxidized states of Mo and W. Yet remarkably, under the same exposure conditions TMD/pyrene remain unperturbed. The current findings demonstrate that pyrene physisorbed on MoS_2_ and WS_2_ acts as an environmental barrier, preventing oxidative surface reactions in the TMDs catalyzed by moisture, air, and assisted by laser irradiation. Raman spectroscopy confirms that the hybrid materials stored under ambient conditions for two years remained structurally unaltered, corroborating the beneficial role of pyrene for not only hindering oxidation but also inhibiting aging.

## 1. Introduction

Transition metal disulfides (TMDs) consist of covalently bound chalcogen–metal–chalcogen layers interacting weakly via van der Waals forces. Wet processing of bulk TMDs is currently the most efficient top-down approach for yielding stable colloidal dispersions of high-quality exfoliated layers [1,2,3,4] enabling easier handling and manipulation. In TMDs, the layer surface is terminated by chalcogen electron lone pairs and thus no dangling bonds are present. Nevertheless, a handicap of the unique structure of monolayer TMDs is the direct exposure of all atoms to the environment, which can induce significant structural modifications, and affect the novel electronic, optical, and mechanical properties [5,6]. Edges and defect sites are susceptible to oxidation, under atmospheric conditions and/or moisture when exposed to light illumination. This forms oxides which can significantly degrade the performance in energy and tribology related applications [7]. Even ‘defect-free’ TMDs oxidize at the basal plane when exposed to atomic oxygen found in low earth orbit environment [8,9,10,11], which can severely impact their use as lubricants in space technology. In addition, the presence of oxygen on the surface of TMDs is a critical issue in micro- and opto-electronic applications, where high electrical conductivity and carrier mobility is required. Chemical vapor deposition grown monolayer MoS_2_ and WS_2_ have been shown to degrade over time even at room temperature, with device currents dropping almost two orders of magnitude after just one month [12]. Even brief ambient exposure (<1 min) was shown to increase contact resistance by over an order of magnitude and decrease intrinsic mobility in MoS_2_ transistors [13].

Different strategies have been adopted to minimize or eliminate these oxidation processes in TMDs, typically through the use of capping layers. These include encapsulation with other monolayer materials such as h-BN [14,15] and graphene [16], or the use of atomic-layer deposition to cover 2D-materials with an oxide layer [17]. However, the former approaches are not very practical or scalable while the latter is limited to nanosheets deposited on a substrate, and thus they are not adapted to solution-based processing. An alternative is the use of polymer encapsulating layers [12] but this requires thick (10–20 nm) polymer layers and the capping layer is difficult to remove without damaging the TMD. It is clear that a simple, scalable, reversible, and solution-based route to environmental protection of TMDs would be highly desirable.

With the aforementioned points in mind and considering the simplicity and reversibility of non-covalent interactions for associating an electron-rich flat all-sp^2^ hybridized carbon species with exfoliated semiconducting 2H-MoS_2_ and 2H-WS_2_, we have developed MoS_2_/pyrene and WS_2_/pyrene ensembles. The π-S interactions between the pyrene moiety and the TMDs were exploited to build the ensembles, avoiding complex and time-consuming methodologies. Importantly such TMDs can be isolated from ambient oxygen environmental conditions, resulting in high stability even after prolonged laser irradiation exposure.

We first focus on the non-covalent modification of TMDs with an electron-donor moiety and examine the optical characteristics and material properties of the corresponding MoS_2_/pyrene and WS_2_/pyrene ensembles. Sample morphology is elucidated by high-resolution scanning transmission electron microscopy (HR-STEM) imaging coupled with electron energy loss spectroscopy (EELS) STEM, while complementary electronic absorption and fluorescence emission spectroscopic assays reveal information related with the photophysical properties of the materials and the existence of electronic interactions in the ground and excited states. Density functional theory (DFT) calculations demonstrate the most favorable pyrene stacking and allow estimation of surface coverage. Then, in order to protect the MoS_2_ and WS_2_ from environmental attack, coverage of TMDs with pyrene as an oxidation resistant species is scrutinized. This new approach has the advantage of non-covalent modification, leaving intact and undisturbed the surface of TMDs, without affecting negatively their electronic and mechanical properties. Additionally, it protects TMDs from oxidation under ambient conditions (air, moisture, light illumination, and aging), without requiring isolation of the material from the environment, minimizing necessity for high-cost equipment and advanced processes for handling the materials.

## 2. Materials and Methods

**General**. All chemicals and solvent were purchased from Sigma Aldrich (Merck KGaA and/or its affiliates, Darmstadt, Germany) and used as received unless otherwise stated. Steady-state UV-Vis electronic absorption spectra were recorded on a PerkinElmer (Lambda 19, PerkinElmer Inc. Madrid, Spain) UV-Vis-Near Infrared (NIR) spectrophotometer. Steady-state emission spectra were recorded on a Fluorolog-3 JobinYvon-Spex spectrofluorometer (model GL3-21, Jobin Yvon Inc. New York, NCY, USA) with 1 nm resolution. Pico-second time-resolved fluorescence spectra were measured by the time-correlated-single-photon-counting (TCSPC, PicoQuant GmbH, Berlin, Germany) method on a Nano-Log spectrofluorometer (Horiba JobinYvon, Bensheim, Germany), using a laser diode as an excitation source (NanoLED, 375 nm, Orlando, FL, USA) and a UV-Vis detector TBX-PMT series (250–850 nm, Horiba JobinYvon, Bensheim, Germany) from Horiba JobinYvon. Lifetimes were evaluated with the DAS6 Fluorescence-Decay Analysis Software (Horiba JobinYvon, Bensheim, Germany). Typically, 100 scans were acquired at 2 cm^−1^ resolution. Micro-Raman scattering measurements were performed at room temperature in the backscattering geometry using a RENISHAW inVia Raman microscope (Renishaw plc, Gloucestershire, UK) equipped with a charge-coupled device (CCD) camera and a Leica microscope. A 2400 lines/mm grating was used for all measurements, providing a spectral resolution of 2 cm^−1^ and an accuracy of ±5 cm^−1^. Measurements were taken with 15 s of exposure times at varying numbers of accumulations. The laser spot was focused on the sample surface using a long working distance 50 × objective (N.A. = 0.35). Raman spectra were collected on numerous spots on the sample and recorded with Peltier cooled CCD camera (Renishaw plc, Gloucestershire, UK). The recorded data were treated with (Renishaw Wire and Origin software (Renishaw plc, Gloucestershire, UK). Thermogravimetric analysis was performed using a TGA Q500 V20.2 Build 27 instrument by TA in a nitrogen (purity > 99.999%) inert atmosphere. The microwave-assisted reaction was performed in a CEM Discover SP reactor employed in open-batch modality. Spatial-resolved EELS measurements were performed on probe-corrected STEM FEI Titan Low-Base 60–300 operating at 80 keV (fitted with an X-FEG^®^ gun, Eindhoven, Netherlands) and Cs-probe corrector (CESCOR from CEOS GmbH, Heidelberg, Germany). EEL spectra were recorded using the spectrum-imaging (SPIM in 2D or spectrum-line (SPLI) in 1D) mode [18,19] in a Gatan GIF Tridiem ESR 865 spectrometer (Pleasanton, CA, USA). The convergent semi-angle was of 25 mrad, the collection semi-angle was 80 mrad and the energy resolution~1.2 eV. All electron microscopy and spectroscopy experiments were conducted at low temperature (−170 °C) to avoid damaging of the pyrene entities. The EELS datasets were denoised with the open-source program Hyperspy by using principal component analysis [19].

**Exfoliation of MoS_2_ and WS_2_**. Bulk TMDs (150–200 mg) were dispersed in chlorosulfonic acid and sonicated for 2 h at room temperature. The solution was left under stirring for a month, occasionally sonicated for 30 s. Afterwards cold water was added to the solution under stirring, drop by drop. This was done carefully since the reaction is exothermic and releases gaseous HCl. Next, the mixture was filtrated on a PTFE filter of 0.2 μm pore-size and washed with excess methanol and acetone. The solid compound was added to N-methyl pyrrolidone and sonicated for 1 h (tip sonication at 30–35% of amplitude (100% of 200 W)). After 3 days the supernatant was taken, filtrated on PTFE filter (0.2 μm pore-size) and washed with a large amount of methanol, acetone and dichloromethane.

**TMD-based nanoensembles 1a and 1b**. A mixture of exfoliated TMDs (20 mg) and pyrene (10 mg) in *N*, *N*-dimethylformamide (DMF) (10 mL) was bath sonicated for 15 min followed by stirring at room temperature with small intervals of sonication for a total period of 36 h. Then, the reaction mixture was filtered over a PTFE filter (100 nm pore size) and the solid residue collected onto the filter was washed with dichloromethane to remove excess free pyrene, furnishing MoS_2_/pyrene 1a and WS_2_/pyrene 1b nanoensembles.

**Density functional calculations**. Density functional theory calculations were performed using the local density approximation (LDA) [20,21,22] (discussion of the functional choice is given at the Supplementary Materials Section). The spin-averaged charge density is fitted to plane waves with an energy cut-off of 150 Ha, while Kohn-Sham wave functions were constructed using localized Cartesian Gaussian orbital functions (50 for Mo, *l* ≤ 3, 28 for S, *l* ≤ 2, 38 for C, *l* ≤ 2, and 12 for H, *l* ≤ 1). Relativistic pseudopotentials generated by Hartwigsen, Goedecker, and Hutter [23] were used, with a finite electron Fermi temperature of 0.04 eV. Absolute energies were converged to better than 10^−5^ Ha. Variable pyrene surface densities were explored using a single pyrene molecule in different hexagonal MoS_2_ supercells, 2 × 2 (Mo_4_S_9_), 3 × 3 (Mo_9_S_18_), 4 × 4 (Mo_16_S_32_), 6 × 6 (Mo_36_S_72_), and 8 × 8 (Mo_64_S_128_), with results referring to the 8 × 8 supercell unless stated otherwise. Gamma-centered k-point meshes generated under the Monkhorst-Pack scheme [24] of 6 × 6 × 1, 4 × 4 × 1, 3 × 3 × 1, 2 × 2 × 1, and 1 × 1 × 1 were used for the increasing supercell sizes. The supercell c-axis was fixed at 31.08 Å to avoid interactions between layers. The binding energy of pyrene with MoS_2_ has been calculated as follows:

*E*_bind_ = (*E*_pyr_ + *E*_MoS2_)–*E*_pyr+MoS2_,

where *E*_bind_ is the binding energy, *E*_pyr+MoS2_ is the energy of the functionalized system, and *E*_pyr_ and *E*_MoS2_ are the energies of the isolated pyrene molecule and MoS_2_ sheet, respectively.

## 3. Results and Discussion

Liquid exfoliated semiconducting 2H-MoS_2_ and 2H-WS_2_ were prepared following literature procedures. Briefly, bulk MoS_2_ and WS_2_ were treated with chlorosulfonic acid to yield exfoliated material of the semiconducting 2H polytype [1]. A solution of TMDs in DMF is mixed with an excess of pyrene for 36 h, during which the pyrene physisorbs onto the surface of the TMDs. The MoS_2_/pyrene and WS_2_/pyrene nanoensembles, referred to hereafter as 1a and 1b, respectively, were obtained free of non-immobilized pyrene by filtering the reaction mixture over a PTFE filter (100 nm pore size) and subsequently washing the solid residue obtained onto the filter with dichloromethane. The process was followed by monitoring the electronic absorption spectrum of the filtrate until no signatures due to pyrene were observed.

The solubility of 1a and 1b was calculated with the aid of UV-Vis spectroscopy by initially estimating the molar absorptivity as 3.24 L g^−1^ cm^−1^ at 400 nm for 1a, 3.04 L g^−1^ cm^−1^ at 465 nm for 1b and then applying the Beer–Lambert law. In general, 1a and 1b ensembles present the best solubility in DMF (100 μg/mL), while they also disperse well in o-DCB (50 μg/mL).

**Figure 1:**
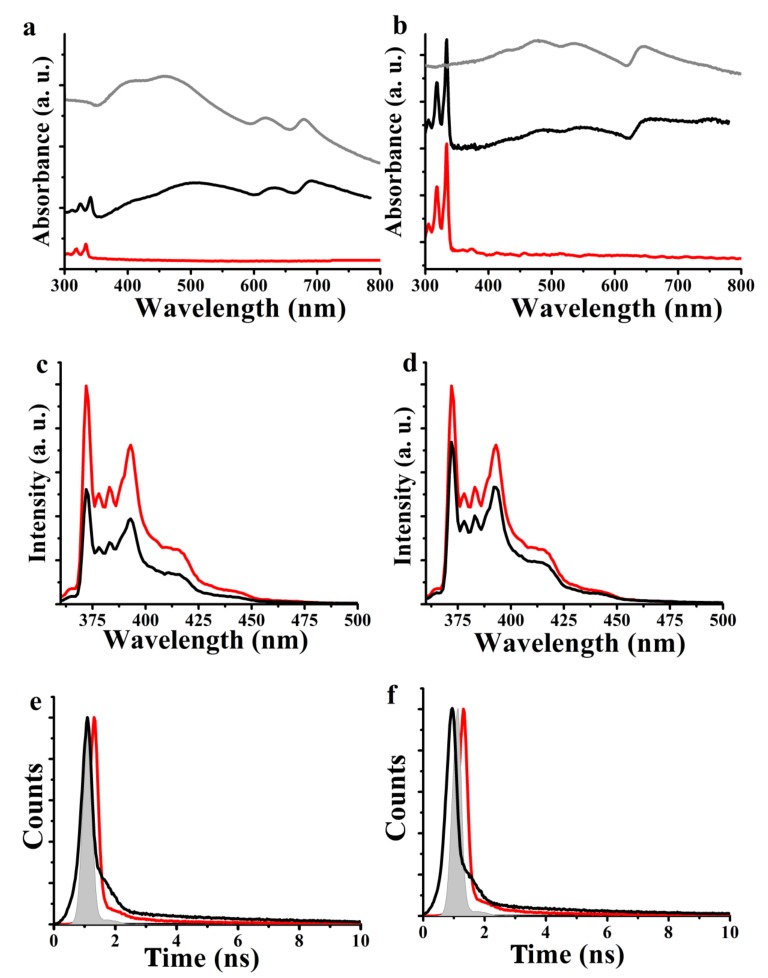
(**a**,**b**) UV-Vis spectra for free pyrene (red) as compared with (**a**) nanoensemble 1a (black line) and exfoliated MoS_2_ (gray line), and (**b**) nanoensemble 1b (black line) and exfoliated WS_2_ (gray line), obtained in DMF. (**c**,**d**) Photoluminescence spectra for free pyrene (red line) as compared with nanoensemble (**c**) 1a (black line), and (**d**) 1b (black line), obtained in DMF upon excitation at 340 nm. (**e**,**f**) Photoluminescence decay time profiles for free pyrene (red) and nanoensemble (**e**) 1a (black line), and (**f**) 1b (black line).

**Figure 2:**
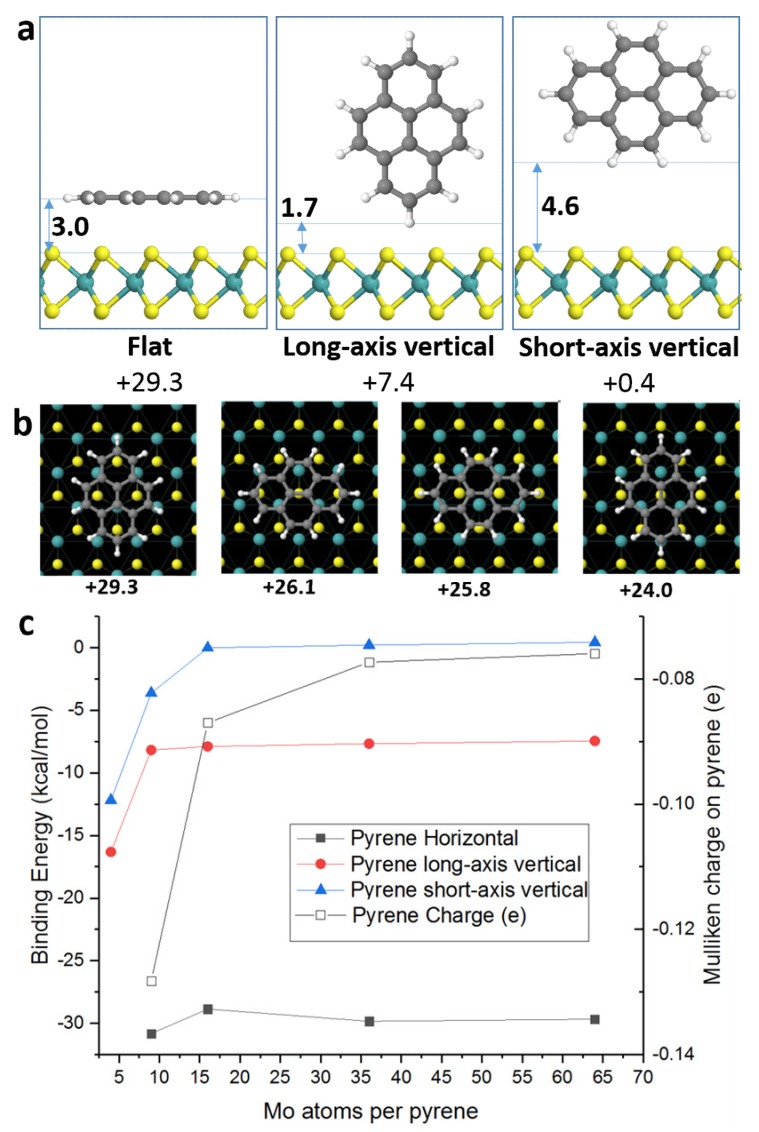
Density functional calculations of pyrene binding to 2H-MoS_2_ showing (**a**) possible stacking orientations of pyrene (binding energies given in kcal/mol, pyrene/MoS_2_ distances in Å), (**b**) variation in binding energy (kcal/mol) for pyrene in parallel-stacked configuration with surface translation. Atom species are Mo (cyan), S (yellow), C (gray), and H (white), and (**c**) variation in binding energy per molecule to MoS_2_ surface for pyrene in different orientations, as a function of surface packing density (filled shapes), right-axis shows Mulliken charge of pyrene in the horizontal configuration (empty squares).

Electronic absorption spectroscopy studies were performed in DMF and absorption bands of pyrene at 315, 330, and 345 nm were detected in both TMD-based 1a and 1b ensembles. Moreover, typical signals of 2H-MoS_2_ at 400, 500, 630, and 690 nm for 1a (Figure 1a) and 2H-WS_2_ at 420, 465, 535, and 640 nm for 1b (Figure 1b) were identified, providing an average of 6–8 layers. These TMD bands were broadened and red-shifted as compared to those of exfoliated 2H-MoS_2_ and 2H-WS_2_, implying ground state electronic interactions between pyrene and the TMDs. Indeed, reversibility is an important parameter and the coated pyrene can be easily removed from the surface of MoS_2_ and WS_2_ by washing the ensembles. UV-Vis absorption spectroscopy of the extensively washed 1a and 1b by dichloromethane revealed only the bands due to MoS_2_ and WS_2_, respectively, without any signature from absorption due to pyrene.

The strong emission of pyrene is a convenient and sensitive probe for identifying itraensemble interactions at the excited states within ensembles 1a and 1b. Photoluminescence of monomeric pyrene with fine structure in the 370–430 nm region was observed for free pyrene upon phtoexcitation at 340 nm in DMF. Then, after adjusting the optical concentration of 1a and 1b with pyrene at the excitation wavelength, the photoluminescence was measured. It was found that the emission from pyrene was appreciably quenched in 1a and 1b as compared to that of free pyrene (Figure 1c,d, respectively). Evidently, the efficient quenching of fluorescence in 1a and 1a implies strong electronic interactions of non-covalently immobilized pyrene with MoS_2_ and WS_2_. In order to further support our results and obtain additional insight on the electronic communication between the individual species within ensembles 1a and 1b, time-resolved fluorescence measurements were performed using the time-correlated-single-photon-counting method. The time profile of the fluorescence decay at 396 nm for the singlet-excited state of free pyrene was monoexponentially fitted with a lifetime of 9.66 ns. Notably, the corresponding fitting for 1a and 1b was biexponential, giving rise to two components, a faster one with lifetime 275 ps, ascribed to efficient interactions of pyrene with MoS_2_ (238 ps for WS_2_), and a slower one with lifetime 5.36 ns attributed to non-interacting pyrene. The quenching rate constant k^S^_q_ for the singlet excited state of pyrene was calculated to be 3.53 × 10^9^ for MoS_2_/pyrene 1a and 4.14 × 10^9^ s^−1^ for WS_2_/pyrene 1b. The corresponding quantum yield Φ^S^_q_ was found to be 0.96 and 0.97, respectively.

We next explore the pyrene/MoS_2_ interaction via density functional theory (DFT) calculations. For comparison we also simulated the two most stable reconstructed 1T-MoS_2_ phases found in Li-exfoliated samples, i.e., with reconstructed Mo-Mo zig-zag surface chains (1T’) and surface trimmers (1T”) [25]. In all cases, the pyrene binding enthalpies are very close (±2.5 kcal/mol) and independent of the underlying MoS_2_ phase, and we report only the 2H-MoS_2_ results here. Figure 2a shows the optimized geometries for pyrene stacked parallel or laterally to the 2H-MoS_2_ surface. The parallel-stacked configuration shows a strong binding of 29.7 kcal/mol whereas the lateral configuration has only 8.3 kcal/mol binding energy. Thus, we conclude that parallel stacking is the most energetically favorable configuration for pyrene on 2H-MoS_2_. We note that the calculated binding energies given here to pristine 2H-MoS_2_ are already quite high. Nonetheless, in the presence of surface defects, we would expect this to increase significantly.

Closer examination of the precise surface positioning of pyrene is shown in Figure 2b, where it can be seen that the most stable position is when the pyrene matches the underlying lattice arrangement, with a sulfur atom positioned beneath each carbon ring. Nonetheless the energy difference remains quite small and at room temperature pyrene is likely to be surface mobile facilitating close-packing arrangements.

To determine the most favorable surface packing density we optimized the three stacking configurations (parallel, long-axis vertical and short-axis vertical) in different size MoS_2_ supercells *n* × *n*, *n* = 2, 3, 4, 6, and 8. The vertical pyrene configurations are always significantly less stable than the parallel packing, even at high surface packing densities where the molecules can π-stack with each other. In the most stable parallel configuration the binding energy increases very slightly at *n* = 3 (by 2 kcal/mol), the maximum possible surface coverage before pyrene overlap. Thus, surface pyrene is most stable at 100% monolayer packing density, i.e., one pyrene per nine Mo atoms.

**Figure 3:**
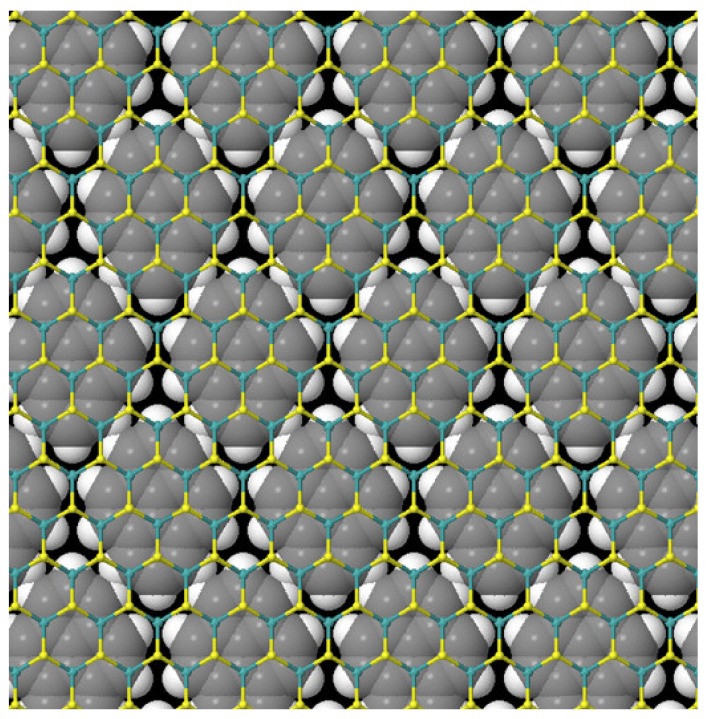
Calculated structure for pyrene in a MoS_2_-3 × 3 surface array at 100% surface coverage, the most stable packing configuration. Pyrene atoms are shown with atomic van der Waals radii, showing the complete surface protective layer. Experimentally we calculate pyrene is forming between two and three layers.

Thermogravimetric analysis (TGA) under nitrogen shows a mass loss of 4.5% and 3.5% in the temperature range 280–550 °C for 1a and 1b, respectively (Supplementary Materials, Figure S1). This mass loss, associated with pyrene decomposition, allowed us to calculate the immobilization of one pyrene unit per every 21 Mo atoms for 1a and per every 17 W atoms for 1b, respectively, representing a rather high coverage. The TGA and DFT results show there would be 100% monolayer coverage for four-layer MoS_2_. From the electronic absorption spectroscopy we estimate an average MoS_2_ flake thickness of six–eight layers and HRTEM confirms that there is typically <10 layers, although the sample is heterogeneous and has some thicker flakes. Thus, we conclude that each MoS_2_ surface is fully saturated with pyrene, with typically two to three layers. This demonstrates the self-organized environmental boundary that pyrene can create on the surface of MoS_2_ (Figure 3). We note that Mulliken analysis suggests quite a large charge transfer of 0.13e from MoS_2_ to pyrene in the ground state (Figure 2c), consistent with the experimental electronic absorption spectroscopy results suggesting ground state electronic interaction between the pyrene and TMDs.

Raman spectroscopy is typically considered a non-invasive characterization technique. However, by varying the incident laser power it is possible to irradiate and possibly modify the sample, while simultaneously obtaining Raman spectra. In this way, we investigated the effect of environmental degradation through local heating and irradiation. Exciting with a 514 nm laser at less than 0.1 mW/4 μm^2^ radiance at ambient conditions, MoS_2_/pyrene 1a and exfoliated MoS_2_ materials all give spectra with the same characteristics. Bands at 378, 405, and 446 cm^−1^ are associated with the E^1^_2g_, A_1g_ and 2LA(M) modes of 2H-MoS_2_, respectively. The absence of characteristic peaks from the metallic polytype 1T-MoS_2_ (J_1_, J_2_, and J_3_) at 150, 225, and 325 cm^−1^ demonstrates the semiconducting nature of MoS_2_ in the 1a ensemble. Edges and basal plane have different intensity in the A_1g_ and E^1^_2g_ modes of the Raman spectrum of the same flake, yet it has been possible to differentiate the effect on the Raman mode between edges, basal plane and ripplocations in single and well-isolated MoS_2_ flakes [26]. Although a detailed Raman spectroscopy analysis goes beyond the main aim of our work, a 6.0 and 2.0 cm^−1^ shift in the E^1^_2g_ and A_1g_ modes for pyrene/MoS_2_ versus exfoliated MoS_2_ are registered. Additionally, A_1g_ shows more than 100% intensity enhancement. However, the E^1^_2g_/A_1g_ intensity ratio is also dependent on other factors e.g., number of layers, thus in our pyrene coated MoS_2_ and WS_2_ materials, in which we have an average of few flakes with very different sizes, such information is problematic. Instead, we focus in the 2LA(M) mode of MoS_2_ commonly related with sulfur vacancies and defects [27,28,29]. Upon 633 nm excitation and due to coupling with the A1 excitonic transition, which produces resonance Raman enhancement of the first and second order vibrational modes, the 2LA(M) mode shows no appreciable changes (Figure S2a), hence proving the non-covalent interactions of pyrene with MoS_2_. Overall, such changes in the Raman modes could be due to the development of electronic interactions between pyrene and MoS_2_ in the ensemble material and/or reduction of the number of MoS_2_ layers resulted by the immobilization of pyrene. Similar is the situation with WS_2_ as we observe a 3.3 cm^−1^ shift in the A_1g_ mode for pyrene/WS_2_ versus the value registered for exfoliated WS_2_. Moreover, changes in the bandwidth have been also observed, e.g., 6 cm^−1^ reduction of the E^1^_2g_ + 2LA(M) modes of pyrene/WS_2_ as compared to exfoliated WS_2_ (Figure S2b).

**Figure 4:**
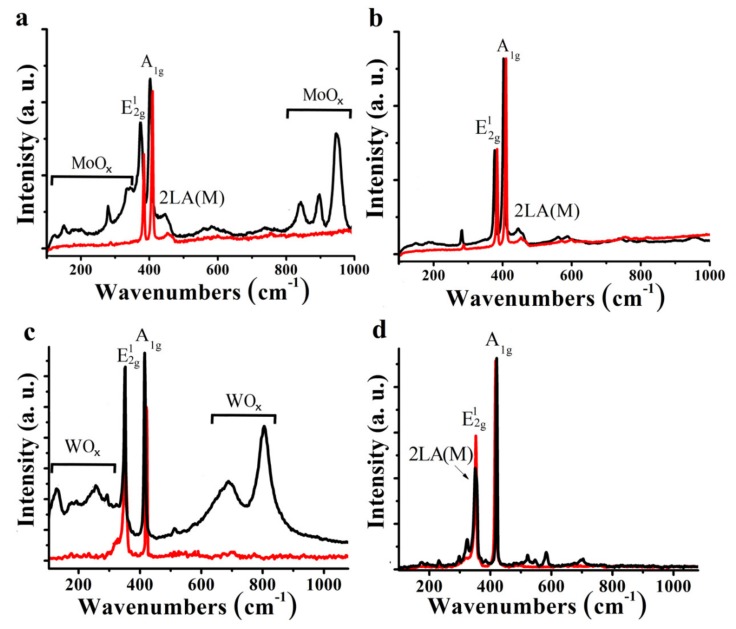
Raman spectra acquired at ambient conditions upon excitation at 514 nm with 0.1 mW/4 μm^2^ (red line) and 0.9 mW/4 μm^2^ (black line) laser radiance for (**a**) exfoliated MoS_2_, (**b**) MoS_2_/pyrene 1a, (**c**) exfoliated WS_2_, and (**d**) WS_2_/pyrene 1b. The E^1^_2g_, A_1g_, and 2LA(M) peaks in the black spectra are downshifted due to laser heating.

However, the situation dramatically changes once the laser radiance is increased to 0.9 mW/4 μm^2^. In addition to a frequency downshift of the previous E^1^_2g_, A_1g_ and 2LA(M) modes, due to laser heating of the sample, new and strong Raman bands appear at a range of energies coming from oxidized states of Mo (Figure 4a). Well-resolved bands appear in the spectrum of exfoliated MoS_2_ at 843, 895, and 948 cm^−1^ due to oxidized Mo [30,31]. In addition, the frequency of broad features at 151, 285, and 340 cm^−1^ are reminiscent of MoO_3_ phases [30,31], however, the deviation from crystalline MoO_3_ in relative peak intensities at higher frequencies (600–1000 cm^−1^) suggests a more disordered structure (MoO_x_). The 948 cm^−1^ peak is likely attributable to either Mo=O or Mo–peroxide stretching modes. These peaks demonstrate the rapid photodegradation of the material upon light irradiation under ambient conditions. Yet remarkably, when MoS_2_/pyrene 1a is subjected to the same exposure conditions (514 nm with 0.9 mW/4 μm^2^) no perturbation of the MoS_2_ is observed, the Raman spectrum retaining its original features (Figure 4b).

A similar situation occurs for WS_2_/pyrene 1b and exfoliated WS_2_. Under resonance conditions with green laser illumination (514 nm, with less than 0.1 mW/4 μm^2^ radiance at ambient conditions) three intense and distinguishable bands at 350, 355, and 418 cm^−1^, due to the 2LA(M), E^1^_2g_ and A_1g_ modes, respectively, are visible (Figure 4c). However, upon increasing the laser radiance to 0.9 mW/4 μm^2^, exfoliated WS_2_ showed additional Raman peaks at 127, 257, and 294 cm^−1^ related to somewhat disordered WO_x_ as well as two sharp peaks at 688 and 805 cm^−1^ (Figure 4c) resembling partially hydrated h-WO_3_ [32]. All those bands associated with WO_x_ species were absent when WS_2_/pyrene 1b was exposed to light irradiation, even at higher laser radiance (Figure 4d). It should also be noted the proportional decrease in the MoS_2_ and WS_2_ characteristic signals E^1^_2g_, A_1g_, and 2LA(M), according to the degree of oxidation. Consequently, pyrene plays an important protecting role of MoS_2_ and WS_2_ from oxidation.

**Figure 5:**
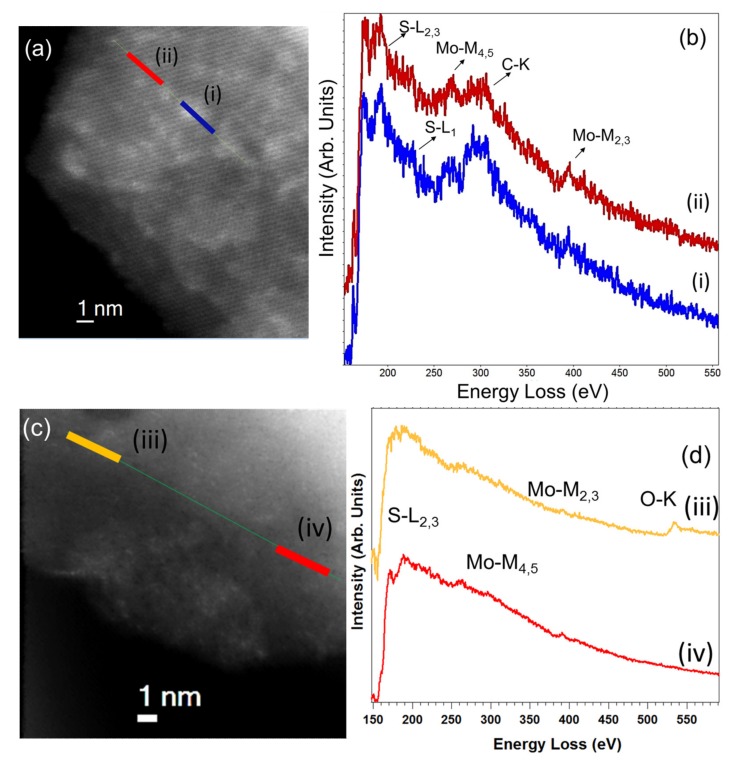
(**a**,**c**) High-angular dark-field (HAADF)-STEM micrographs of MoS_2_/pyrene 1a and exfoliated MoS_2_ flakes, respectively. Two electron energy loss spectroscopy (EELS) line spectra were recorded on these flakes, see the green lines marked in Figure 5a,c. (**b**) Two EEL spectra corresponding to the sum of seven spectra recorded in each of the two areas highlighted in red (i) and blue (ii) in Figure 5a, respectively. Carbon, corresponding to pyrene, sulfur, and molybdenum (associated to MoS_2_) are detected in these spectra. (**d**) Two EEL spectra corresponding to the addition of eight spectra collected in each of the two regions highlighted in orange (iii) and red (iv) in Figure 5c, respectively. The presence of oxygen denotes the clear oxidation of this MoS_2_ flake, see text for details.

In order to investigate in more detail at the local scale the pyrene immobilization onto the TMD materials, a different TEM analysis was performed. These kinds of analyses are required to have access to this structural and chemical information at the (sub-)nanometer level [27,33,34,35,36]. Figure 5a,c shows STEM high-angular dark-field (HAADF) images of two flakes of MoS_2_/pyrene 1a (Figure 5a) and exfoliated MoS_2_ (Figure 5c), both after laser-irradiation (0.9 mW/4 μm^2^) at ambient conditions. Two EEL line spectra (LS) were acquired following the green lines marked on the micrographs. For each sample two different regions, marked as (i) and (ii) in each of the images were selected. The spectra in Figure 5b for 1a show S-L_2,3_, Mo-M_4,5_, C-K, and Mo-M_2,3_ edges. The Mo and S edges correspond to the MoS_2_ flake and the carbon content confirms the presence of the pyrene moieties [28,35,36]. Remarkably, examining close to fifteen different areas of different flakes, no oxygen was detected in MoS_2_/pyrene ensemble 1a. However, this was not the case for exfoliated MoS_2_ without any pyrene immobilized on it, where oxygen was easily detected (Figure 5c,d). This oxygen (see Figure 5d-iii) corresponds to the oxidation of the MoS_2_ flake, where a mix of molybdenum oxide and molybdenum disulfide can be observed. This conclusion can be clearly inferred from a comparison of the S-L_2,3_ edge in the two different selected regions of the LS of Figure 5c. The S-L_2,3_ edge of the EEL spectrum in Figure 5d-iii is similar to that obtained by others investigating mixed MoO_x_-MoS_2_ phases in nanomaterials [37]. Similar imaging assays have been performed for WS_2_/pyrene 1b (Figure S3).

Shedding light on the oxidation mechanism, initially, oxygen from the air is physisorbed onto the exfoliated MoS_2_ and WS_2_. Upon laser irradiation, the surface of the TMDs is damaged, with defective sites created and stabilized by chemisorbed oxygen. Sulfur atoms may leave the lattice and be substituted by oxygen [38,39,40], resulting in structural deformation, as well as hole formation whose edges can become oxygen saturated. Localized heating, due to Raman laser irradiation conditions, further damages the chemisorbed TMDs surface, generating abundant structural defects and etching the surface. The result of such processes are visible in the Raman spectra of exfoliated MoS_2_ and WS_2_ after prolonged irradiation period, where not only new bands due to MoO_x_ and WO_x_ appear, while the intensity of the characteristic A_1g_, E^1^_2g_, and 2LA(M) modes decreases.

At the same time, interaction with surface water and subsequent degradation is an important process that can also be blocked by surface pyrene. The surface of TMDs is intrinsically mildly hydrophilic, due to both the transition metal and the chalcogen atoms. Moreover, doping TMDs with oxygen results in a more hydrophilic surface [41], susceptible to hydrogen-bonding interactions with moisture from air. Then, the presence of Mo-edge-defected MoS_2_ structures (or W for WS_2_) results in moisture air-induced transformations of sulfur to sulfate species, eventually leading to MoO_x_ and WO_x_ accompanied by the liberation of sulfuric acid via a series of oxidation and replacement reactions [42]. Conversely, adsorption of hydrophobic hydrocarbons onto TMDs renders the surface layer hydrophobic [43]. Hence, physisorption of pyrene onto MoS_2_ and WS_2_ acts as barrier, insulating TMDs from the outer environment and prevents the initiation of surface reactions towards vacancies creation, sulfate/sulfuric acid species generation, substitution of sulfur by oxygen, etc., which are catalyzed by humid air and assisted by laser irradiation. Furthermore, pyrene is likely to bind even more strongly to defective sites than pristine basal material, further driving its barrier capability.

**Figure 6:**
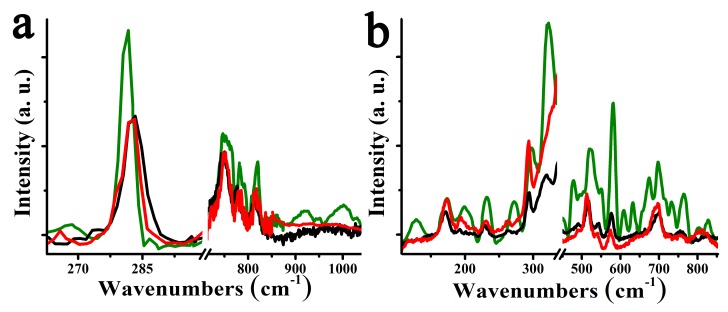
Raman spectra at 514 nm with 0.1 mW/4 μm^2^ laser radiance, for (**a**) fresh exfoliated MoS_2_ (black), aged exfoliated MoS_2_ (green) and aged MoS_2_/pyrene 1a (red), and (**b**) fresh exfoliated WS_2_ (black), aged exfoliated WS_2_ (green) and aged WS_2_/pyrene 1b (red). The region showing the characteristic Raman bands due to MoS_2_ and WS_2_ have deliberately been omitted to allow better observation of the bands due to MoO_x_ and WO_x_.

Exfoliated MoS_2_ and WS_2_ stored in the dark for more than two years in powder form and in the presence of air show a non-depreciated enhancement in the oxidation Raman bands. In fact, WS_2_ was found to be more susceptible to oxidation as compared to MoS_2_, which can be rationalized by considering the higher number of electrons present in W as compared to those in Mo. Interestingly, those oxidation features are discernible, but to a lower extent (Figure 6), even in freshly exfoliated TMDs and according to a couple of very recent articles this is due to defective passivation with oxygen under air exposure in ambient conditions [44,45]. Then, coated pyrene plays a protective role, as after two years no obvious change in the Raman spectra for 1a and 1b is observed (Figure 6). In sharp contrast, freshly exfoliated TMDs, without coated pyrene, show an appreciable oxidation effect after two years in the dark and in the presence of air. First, enhancement of the oxidation Raman bands for around 70% and more than 100% in MoS_2_ and WS_2_ respectively, is identified. Then, more relevant is the presence of new features at 921 and 1002 cm^−1^ due to oxidation in the aged exfoliated MoS_2_ and 233 and 765 cm^−1^ in the aged exfoliated WS_2_. In addition, the shift for some of the oxidation Raman modes, such as 1 and 4 cm^−1^ at 282 and 813 cm^−1^ in fresh MoS_2_ and 3, 8, and 10 cm^−1^ at 514, 754, and 764 cm^−1^ in fresh WS_2_, clearly show evolution in the oxidative species. Overall, we note that materials kept for more than two years remain structurally unaltered, as demonstrated in the Raman spectra of 1a and 1b (Figure 6), showing that pyrene not only blocks oxidation but also prevents aging of TMDs [12,16].

## 4. Conclusions

Pyrene has been demonstrated to bind to the basal plane of MoS_2_ and WS_2_ via multiple π-S interactions, yielding a close-packed continuous surface coverage with significant ground-state charge-transfer. This simple non-destructive and reversible process has the advantage of non-covalent modification, leaving intact and undisturbed the surface of TMDs, without permanently affecting their electronic and mechanical properties, in contrast with covalent bonding incorporation of other photoactive species [29,46,47,48,49,50].

The resultant dense pyrene coating has been shown using Raman spectroscopy to shield the surface of TMDs from environmental degradation under ambient conditions (air, moisture, and light illumination), without requiring isolation of the material from the environment, minimizing necessity for high-cost equipment and advanced processes for handling the materials. The additional beneficial role of pyrene for inhibiting TMD aging was demonstrated via unaltered Raman spectroscopy of protected samples stored under ambient conditions for two years. This simple and effective oxidation resistance route should also be applicable to other TMDs and 2D-layered materials, such as HfSe_2_ which is known to be particularly susceptible to oxidation [51].

## Supplementary Materials

The following are available online at https://www.mdpi.com/2079-4991/10/2/363/s1, Figure S1: TGA of (a) 1a (red line) as compared with exfoliated MoS_2_ of the semiconducting polytype (black line), (b) and 1b (red line) as compared with exfoliated WS_2_ of the semiconducting polytype (black line) under nitrogen atmosphere.; Figure S2: Raman spectra (a) at 633 nm with 0.1 mW/4 μm^2^ laser radiance, for exfoliated MoS_2_ (black) and MoS_2_/pyrene 1b (red), and (b) at 514 nm with 0.1 mW/4 μm^2^ laser radiance, for exfoliated WS_2_ (black) and WS_2_/pyrene 1b (red); Figure S3: (a,c) HAADF-STEM micrographs of WS_2_/pyrene 1b and exfoliated WS_2_ flakes, respectively. An EELS spectrum-image and an EELS spectrum-line have been recorded on these respective flakes, see the green line marked in Figure S3c. (b) Two EEL spectra corresponding to the sum of fourteen spectra recorded in each of the two areas highlighted in red (i) and blue (ii) in Figure S3a, respectively. Carbon, corresponding to pyrene and sulfur (associated to WS_2_) are detected in these spectra. (d) Two EEL spectra corresponding to the addition of twelve spectra collected in each of the two regions highlighted in orange (iii) and red (iv) in Figure S3c, respectively. The presence of oxygen denotes the clear oxidation of this WS_2_ flake, as it was the case for MoS_2_ (see Figure 5 in the paper); Table S1: Comparison of calculated structural parameters and cohesion energies for 2H-MoS_2_ with different approximations and the corresponding experimental data (Exp.). The LDA optimisations have been performed allowing atoms and lattice parameter to change in a hexagonal lattice using a 24×24×12 MP *k*-point grid. The cohesion energy (∆E_coh_) is taken as the energy difference between isolated S and Mo atoms and MoS_2_ normalized per atom. For the isolated atoms spin-polarised calculations with a single *k*-point at Г have been performed, while MoS_2_ has been calculated with a spin-averaged approach; Table S2: Binding energies (eV) for the adsorption of benzene and naphthalene on the surface of MoS_2_ calculated using different methods. Our calculations have been performed on an 8×8 supercell of MoS_2_. The binding energy is taken as the difference in energy between the combined system (MoS_2_+ molecule) and the energy of the separate components. Both MoS_2_ and the isolated molecules have been calculated using a single *k*-point at Г.
