# Supplementary material for: Pyrene Coating Transition Metal Disulfides as Protection from Photooxidation and Environmental Aging
Source: Nanomaterials (Basel). 2020 Feb 19;10(2):363. doi: 10.3390/nano10020363 (PMC7075307; doi:10.3390/nano10020363)
Supplement: Supplementary file 1 [file nanomaterials-10-00363-s001.pdf]

Article

Pyrene Coating Transition Metal Disulfides as Protection from Photooxidation and Environmental Aging

Ruben Canton-Vitoria [1,†], Yuman Sayed-Ahmad-Baraza [2,†], Bernard Humbert [2], Raul Arenal [3,4,5,*], Christopher P. Ewels [2,*] and Nikos Tagmatarchis [1,*]

1 Theoretical and Physical Chemistry Institute, National Hellenic Research Foundation, 48 Vassileos Constantinou Avenue, 11635 Athens, Greece.
2 Institut des Materiaux Jean Rouxel (IMN), UMR6502 CNRS, Universite de Nantes, 2 Rue de la Houssiniere, BP32229, 44322 Nantes, France.
3 Laboratorio de Microscopias Avanzadas, Instituto de Nanociencia de Aragon, Universidad de Zaragoza, 50018 Zaragoza, Spain
4 ARAID Foundation, 50018 Zaragoza, Spain
5 Instituto de Ciencias de Materiales de Aragon, CSIC-U. Zaragoza, 50009 Zaragoza, Spain
* Correspondence: arenal@unizar.es (R.A.); chris.ewels@cnrs-imn.fr (C.P.E.); tagmatar@eie.gr (N.T.)
† These authors contributed equally to this work.

Received: 29 January 2020; Accepted: 14 February 2020; Published: date

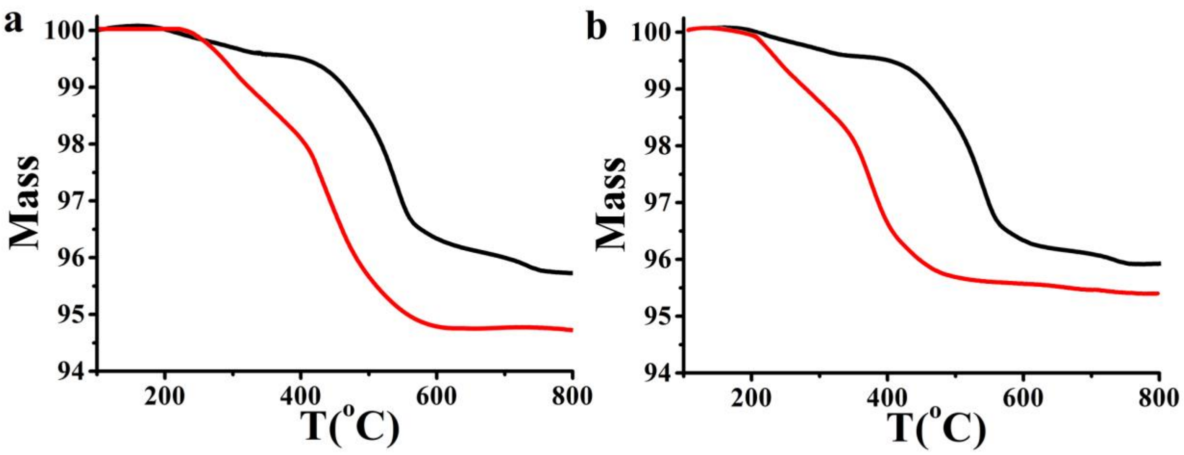

Figure S1. TGA of (**a**) **1a** (red line) as compared with exfoliated MoS_2 of the semiconducting polytype (black line), (**b**) and **1b** (red line) as compared with exfoliated WS_2 of the semiconducting polytype (black line) under nitrogen atmosphere.

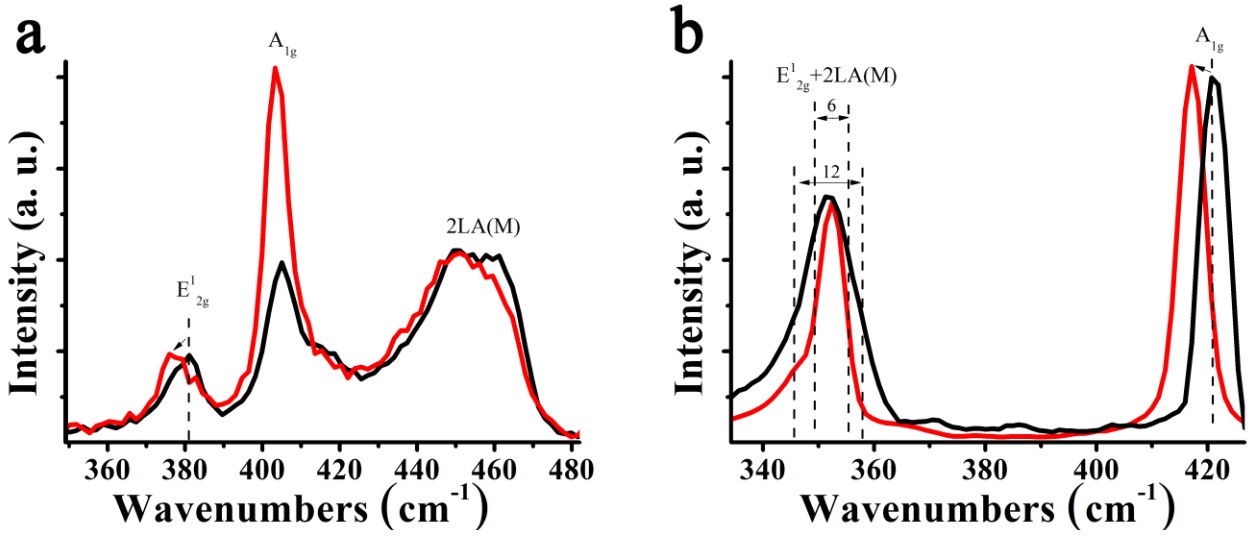

Figure S2. Raman spectra (**a**) at 633 nm with 0.1 mW/4 μm² laser radiance, for exfoliated MoS_2 (black) and MoS_2/pyrene **1b** (red), and (**b**) at 514 nm with 0.1 mW/4 μm² laser radiance, for exfoliated WS_2 (black) and WS_2/pyrene **1b** (red).

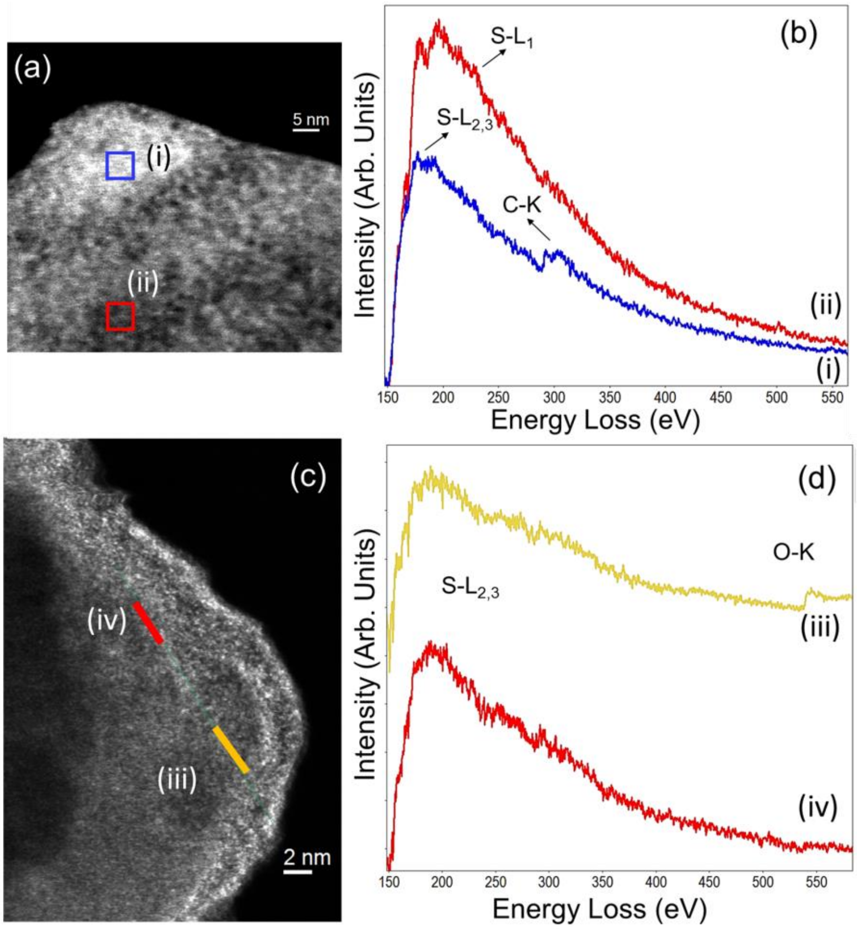

Figure S3. (**a,c**) HAADF-STEM micrographs of WS**2**/pyrene **1b** and exfoliated WS_2 flakes, respectively. An EELS spectrum-image and an EELS spectrum-line have been recorded on these respective flakes, see the green line marked in Figure S3c. (**b**) Two EEL spectra corresponding to the sum of fourteen spectra recorded in each of the two areas highlighted in red (i) and blue (ii) in Figure S3a, respectively. Carbon, corresponding to pyrene and sulfur (associated to WS_2) are detected in these spectra. (**d**) Two EEL spectra corresponding to the addition of twelve spectra collected in each of the two regions highlighted in orange (iii) and red (iv) in Figure S3c, respectively. The presence of oxygen denotes the clear oxidation of this WS_2 flake, as it was the case for MoS_2 (see Fig. 5 in the paper).

Benchmarking the Local Density Approximation for MoS_2

Although the LDA does not include any dispersion corrections, this is to some extent compensated by the over-binding present in LDA. The LDA also includes explicitly all weak covalent interaction between surface adsorbed species and the underlying substrate. This effect has been quantified, for example, in studies of interlayer binding in graphite, where half of the interaction is found to be non-dispersive, and where the LDA reproduces with reasonable accuracy both the interlayer binding energy and interlayer spacing.[1] In addition, the adsorption of several polyaromatic compounds on graphene has been studied with different functionals, and LDA presented reasonable results for planar stacked configurations.[2] Although dispersion corrections were considered important to obtain accurate binding energies in this study, it was found that inclusion of the dispersive interactions did not change the shape of the interaction energy surfaces or the value of the energy barriers for the motion of polyaromatic molecules on graphene. Equally benzene-benzene interaction is found to be structurally correct even if some overbinding is predicted compared to higher levels of theory.[3]

Concerning the use of LDA to describe MoS_2 we have calculated the structural parameters and cohesion energies of bulk 2H-MoS_2 and compared our results with experimental data and with results obtained using other theoretical methods. In particular, we compared our LDA results to literature values obtained by the Tkatchenko-Scheffler (TS) van der Waals corrected method (including the self-consistently screened SCS variant), the GGA PBE and the Grimme-D2 dispersion corrected GGA PBE methods.

Table S1. Comparison of calculated structural parameters and cohesion energies for 2H-MoS_2 with different approximations and the corresponding experimental data (Exp.). The LDA optimisations have been performed allowing atoms and lattice parameter to change in a hexagonal lattice using a 24x24x12 MP *k*-point grid. The cohesion energy (ΔE_{coh}) is taken as the energy difference between isolated S and Mo atoms and MoS_2 normalized per atom. For the isolated atoms spin-polarised calculations with a single *k*-point at Γ have been performed, while MoS_2 has been calculated with a spin-averaged approach.

Parameters	Exp.	LDA (our work)	PBE[4]	PBE +D2[4]	TS[5]	TS +SCS[5]
Lattice *a* (Å)	3.160[6]	3.13	3.18	3.19	3.16	3.16
Lattice *c* (Å)	12.294[6]	12.04	14.68	12.42	12.03	12.01
V (Å^3)	106.3[6]	102.27	128.8	109.4	103.9	104.0
c/*a*	3.891[6]	3.85	4.616	3.893	3.81	3.79
ΔE_{coh} (eV/atom)	5.18[7]	7.11	5.12	5.37	5.33	5.31

As can be seen in **Error! Reference source not found.**, the LDA results are, generally, in good agreement with the experimental data, and are in the same error range as the dispersion corrected results. The LDA predicts a smaller *a* lattice parameter than experiment, while the opposite occurs for the PBE calculation, and only the TS calculations show the correct value. Notably, the *c* lattice parameter, related with the interlayer interaction, is remarkably well predicted with LDA, as well as the cell volume (V) and the *c*/*a* ratio. The PBE functional without dispersion corrections is the approximation that shows worst results for these parameters.

The exception is the cohesive energy, for which LDA presents a significant over-binding, presumably due to a very poor description of the isolated atoms in LDA, and the known over-binding tendency of this approximation.

We expect that LDA would also be a relatively good approximation for the study of the interaction of MoS_2 with aromatic molecules, despite the lack of dispersion corrections. In order to

check that, we have calculated the binding energy for the adsorption of benzene and naphthalene on the surface of MoS_2, and compared the resulting values with those obtained using other methods that include Van der Waals (VdW) corrections (**Error! Reference source not found.**2).

Table S2. Binding energies (eV) for the adsorption of benzene and naphthalene on the surface of MoS_2 calculated using different methods. Our calculations have been performed on an 8x8 supercell of MoS_2. The binding energy is taken as the difference in energy between the combined system (MoS_2+ molecule) and the energy of the separate components. Both MoS_2 and the isolated molecules have been calculated using a single *k*-point at Γ.

Molecules	LDA (our work)	RPBE-vdW[8]	revPBE vdW-DF[8]
Benzene	-0.66	-0.51	-0.47
Naphthalene	-0.92	-0.75	-0.70

The LDA results are reasonable in comparison with the other methods that include VdW corrections. Even without dispersion corrections, the LDA is still presenting a slight over-binding. In any case, the LDA calculations reproduces the trends predicted by the VdW corrected methods.

References

1. J.-C. Charlier, X. Gonze, and J.-P. Michenaud, "Graphite interplanar bonding: Electronic delocalization and van der Waals interaction", *Europhys. Lett.*, 28 (6), 403–408 (1994).
2. P. Nachtigall and C. O. Arean, "Study of polycyclic aromatic hydrocarbons adsorbed on graphene using density functional theory with empirical dispersion correction", *Phys. Chem. Chem. Phys.*, 12 (24), 6307 (2010).
3. Yaya, E. K. Tiburu, B. Onwona-Agyeman, D. Dodoo-Arhin, and J. K. Efavi, "A comparative study of DFT/LDA with higher levels of theory on π-π interactions: A typical case for the benzene dimer", *J. Computations & Modelling*, 4 (3), 27-42 (2014).
4. T. Bučko, J. Hafner, S. Lebègue, and J. G. Ángyán, "Improved description of the structure of molecular and layered crystals: Ab initio DFT calculations with van der Waals corrections", *J. Phys. Chem. A*, 114 (43), 11814-11824 (2010).
5. T. Bučko, S. Lebègue, J. Hafner, and J. G. Ángyán, "Tkatchenko-Scheffler van der Waals correction method with and without self-consistent screening applied to solids", *Phys. Rev. B*, 87 (6), 064110 (2013).
6. K. D. Bronsema, J. L. D. Boer, and F. Jellinek, "On the structure of molybdenum diselenide and disulfide", *Z. Für Anorg. Allg. Chem.*, 540 (9–10), 15–17 (1986).
7. P. Raybaud, G. Kresse, J. Hafner, and H. Toulhoat, "Ab initio density functional studies of transition-metal sulphides: I. Crystal structure and cohesive properties", *J. Phys. Condens. Matter*, 9 (50), 11085–11106 (1997).
8. P. G. Moses, J. J. Mortensen, B. Lundqvist, J. K. Norskov, "Density functional study of the adsorption and van der Waals binding of aromatic and conjugated compounds on the basal plane of MoS_2", *J. Chem. Phys.*, 130 (10), 104709 (2009).
